# Ovarian steroids regulate tachykinin and tachykinin receptor gene expression in the mouse uterus

**DOI:** 10.1186/1477-7827-7-77

**Published:** 2009-07-23

**Authors:** Francisco M Pinto, C Oscar Pintado, Jocelyn N Pennefather, Eva Patak, Luz Candenas

**Affiliations:** 1 Instituto de Investigaciones Químicas, CSIC, Avda. Americo Vespucio 49, 41092, Sevilla, Spain; 2 Centro de Producción y Experimentación Animal, Universidad de Sevilla, Sevilla, Spain; 3 Department of Pharmaceutical Biology, Monash University, Parkville, Victoria 3052, Australia; 4 Department of Anaesthetics, Royal Women's Hospital, Carlton, Victoria 3051, Australia

## Abstract

**Background:**

In the mouse uterus, pregnancy is accompanied by changes in tachykinin and tachykinin receptor gene expression and in the uterotonic effects of endogenous tachykinins. In this study we have investigated whether changes in tachykinin expression and responses are a result of changes in ovarian steroid levels.

**Methods:**

We quantified the mRNAs of tachykinins and tachykinin receptors in uteri from ovariectomized mice and studied their regulation in response to estrogen and progesterone using real-time quantitative RT-PCR. Early (3 h) and late (24 h) responses to estrogen were evaluated and the participation of the estrogen receptors (ER), ERalpha and ERbeta, was analyzed by treating mice with propylpyrazole triol, a selective ERalpha agonist, or diarylpropionitrile, a selective agonist of ERbeta.

**Results:**

All genes encoding tachykinins (Tac1, Tac2 and Tac4) and tachykinin receptors (Tacr1, Tacr2 and Tacr3) were expressed in uteri from ovariectomized mice. Estrogen increased Tac1 and Tacr1 mRNA after 3 h and decreased Tac1 and Tac4 expression after 24 h. Tac2 and Tacr3 mRNA levels were decreased by estrogen at both 3 and 24 h. Most effects of estrogen were also observed in animals treated with propylpyrazole triol. Progesterone treatment increased the levels of Tac2.

**Conclusion:**

These results show that the expression of tachykinins and their receptors in the mouse uterus is tightly and differentially regulated by ovarian steroids. Estrogen effects are mainly mediated by ERalpha supporting an essential role for this estrogen receptor in the regulation of the tachykinergic system in the mouse uterus.

## Background

Uterine function is tightly controlled by ovarian steroids [1-5]. The physiological responses to acute estrogen (E_2_) occur in two temporally distinct steps. Early responses appear within the first 2–3 h after E_2 _administration while late responses are observed 16–24 h after E_2_, with each step involving activation of distinct sets of genes [4,5]. E_2 _and progesterone (P_4_) exert their effects by binding to specific transcription factor receptors, the estrogen receptors α (ERα) and β (ERβ) and the progesterone receptors (PR A and B), respectively [2-7]. In addition to these classical genotropic effects, E_2 _and P_4 _activate extranuclear, non-genomic signaling cascades [8,9]. It is still unclear whether non-genomic effects are mediated by membrane receptors distinct from the nuclear receptors, or additionally involve activation of classical ERs or PRs located outside the nucleus [4-9].

Accumulating evidence suggests that tachykinins (TKs) play a role in the regulation of uterine function [3,10]. TKs comprise a family of peptides, which in mammals includes substance P (SP), neurokinin A (NKA) neurokinin B (NKB) and hemokinin-1 (HK) [11-17]. In mice, SP and NKA are encoded by the *Tac1 *gene (accession number NM_009311), NKB by the *Tac2 *gene (accession number ENSMUST00000026466) and HK by the *Tac4 *gene (accession number NM_053093) [10-14]. Their biological effects are mediated by receptors belonging to the family of G protein-coupled receptors. Three different tachykinin receptors are currently recognized, namely the NK_1 _receptor (NK_1_R), the NK_2 _receptor (NK_2_R) and the NK_3 _receptor (NK_3_R) which, in mice, are encoded by the *Tacr1*(accession number NM_009313), *Tacr2 *(accession number NM_009314) and *Tacr3 *(accession number NM_021382) genes, respectively [10-17]. The endogenous tachykinins bind with differing affinities to each of the tachykinin receptors. Thus, the NK_1_R is activated preferentially by SP and HK, the NK_2_R by NKA and the NK_3_R by NKB [16,17].

Tachykinins modulate reproductive function at both central and peripheral levels [18-35]. In the central nervous system, SP, NKA and NKB are expressed in subpopulations of hypothalamic and pituitary neurons where they influence, and are influenced by, gonadotropin and gonadal steroid levels [18-22] and modulate the synthesis and/or secretion of oxytocin [28]. In the reproductive tract, these peptides are expressed in sensory nerves and in non-neuronal cells within the placenta, the ovary, the uterus [3,10,24-26,29-34], the testes and the prostate [3,36]. They are also present in other reproductive cells including corpora lutea, oocytes and spermatozoa [33,35,37].

We have previously found that all genes encoding TKs and their receptors are expressed in the mammalian uteri and that their expression and function vary during the ovarian cycle, throughout pregnancy and with age, [3,10,29,30,38,39]. In late pregnant and non-pregnant women, in rats, and in late pregnant mice, TKs induce myometrial contractions that are mediated mainly by the NK_2_R with minor participation of NK_1 _and NK_3 _receptors [10,29,30,39]. In contrast, in non-pregnant mice, the predominant receptor mediating myometrial contractions is the NK_1_R, with NK_2_R playing a minor role [10,29,39]. The reasons for this difference between species remain unclear. Indeed, little is known about the physiological mechanisms that regulate the expression of TKs and their receptors in mouse uterus and in particular the influence of ovarian steroids upon their expression.

In this study, we have analyzed the direct effects of the ovarian steroids E_2 _and P_4 _on the expression of *Tac1, Tac2, Tac4, Tacr1, Tacr2 *and *Tacr3 *in uteri from ovariectomized mice. In addition, we have investigated the effects of the selective agonist of the estrogen receptor α (ERα) propylpyrazole triol (PPT) [7,40], and the selective agonist of the estrogen receptor β (ERβ) diarylpropionitrile (DPN) [7], in order to define the roles played by ERα and ERβ in the regulation of TK and TK receptor gene expression. We have also analyzed the effects of estren, a selective activator of non-genomic E_2 _signaling pathways [8]. Because E_2_-induced changes in gene expression occur as early or late responses [4,5], animals were treated with E_2_, PPT, DPN or estren for either 3 or 24 h.

## Methods

### Animals and treatments

Ethical approval for this study was obtained from Consejo Superior de Investigaciones Científicas (Spain). Virgin female Swiss mice (7–9 weeks, 20–25 g) were purchased from Charles River Laboratories (Barcelona, Spain), housed at 22°C under controlled lighting (12:12-h light/dark cycle) and provided with food and water *ad libitum*. Animals were bilaterally ovariectomized under avertin anesthesia (250 mg/Kg, i.p.). Fifteen days later, they were left untreated (control, untreated mice) or treated s.c. with a) E_2 _(17β-estradiol benzoate, Sigma, 1 μg/mouse); b) the selective ERα agonist propylpyrazole triol (PPT, Tocris, Ellisville, MO, 75 μg/mouse); c) the selective ERβ agonist diarylpropionitrile (DPN, Tocris, 100 μg/mouse); d) estren (Steraloids Newport, RI, 300 μg/mouse) or e) the same volume of vehicle (olive oil, 100 μl) (control, vehicle-treated mice). In all cases, uteri were collected 3 and 24 h after the injection. In a second set of experiments, mice were treated with a) P_4 _(Sigma, 2 mg/mouse per day for 2 days) or its vehicle, and uteri collected 24 h after the last injection; b) a single injection of E_2 _(1 μg/mouse) or its vehicle, followed 24 h later by P_4 _(2 mg/mouse per day for 2 days) or its vehicle and uteri collected 24 h after the last injection; c) a single injection of E_2 _(1 μg/mouse) or its vehicle and uteri collected 72 h after treatment. The pharmacological doses of steroid receptor ligands used in the present experiments were based on previously published studies [2,4-8,39].

### Real-time RT-PCR

Uterine samples were rapidly immersed in RNAlater (Ambion, Huntingdon, UK) and stored at -80°C until use. Total RNA isolation and cDNA synthesis were carried out as previously described [29]. The cDNA samples were amplified by PCR using specific oligonucleotide primer pairs (Table 1) designed with the software Primer 3 and purchased from Sigma-Genosys (Cambridge, UK) [29]. A specific probe (n° 88, Home probe Library, Exiqon, Vedbaek, Denmark), chosen with the program Probefinder assay design (Exiqon), was used to detect the *Tac1 *transcript (88 bp) since this gene was present in faint amounts in uteri from pregnant and non-pregnant mice [29]. With this approach, we found that *Tac1 *mRNA levels were 4.5- and 3.0-fold higher in untreated ovariectomized mice, than in estrus and diestrus virgin mice, respectively (unpublished observations). The specific primer pair shown in Table 1 was employed in subsequent experiments for *Tac1 *detection. Table 1 also shows the primers used to amplify β-actin (*Actb*), protein phosphatase 1 catalytic subunit beta-isoform (*Ppp1cb*), glyceraldehyde-3-phosphate-dehydrogenase (*Gapdh*) and polymerase (RNA) II (DNA directed) polypeptide A (*Polr2a*), which were chosen as housekeeping genes for normalizing the PCR data on the basis of previous studies in the mouse and rat uterus [29,36,37].

**Table 1:** Nucleotide sequence of the specific primers used in PCR amplifications.

Gene	Primers	Sense	Product Size
*Tac1*	5'-GGCCAAGGAGAGCAAAGA-3'	F	88 bp
	5'-CGAGGATTTTCATGTTCGATT-3'	R	
*Tac2*	5'-TCTGGAAGGATTGCTGAAAGTG-3'	F	302 bp
	5'-GTAGGGAAGGGAGCCAACAG-3'	R	
*Tac4*	5'-GTAGCTTCCTCAGCCATGCAG-3	F	186 bp
	5'-CCGCCCCCAAATACAATACA-3'	R	
*Tacr1*	5'-GCCAGAACATCCCAACAGG-3'	F	223 bp
	5'-GGCGAAGGTACACACAACCA-3'	R	
*Tacr2*	5'-TGGTACTGGTGGTGGTGACATT-3'	F	251 bp
	5'-CCTGTCTTCCTCGGTTGGTG-3'	R	
*Tacr3*	5'-CCAACTACTGCCGCTTCCA-3'	F	272 bp
	5'-GAAATGTTGCTTGGGACCTTCT-3'	R	
*Actb*	5'-TCCCTGGAGAAGAGCTACGA-3'	F	362 bp
	5'-ATCTGCTGGAAGGTGGACAG-3'	R	
*Gapdh*	5'-CAATGCCTCCTGCACCAC-3'	F	350 bp
	5'-CCTGCTTCACCACCTTCTTG-3'	R	
*Ppp1cb*	5'-AACCATGAGTGTGCTAGCATCA-3'	F	472 bp
	5'-CACCAGCATTGTCAAACTCGCC-3'	R	
*Polr2a*	5'-CGTTTCCATCCTAAGCCCAGT-3'	F	260 bp
	5'-ATCTCTGCCCGTGTTTCCAG-3'	R	

Shown are the structures of forward (F) and reverse (R) primers of indicated target genes and the size expected for each PCR-amplified product. Primers for *Actb *(β-actin), *Gapdh*, *Ppp1cb and Polr2a*, used as housekeeping genes, are also shown.

Real-time quantitative PCR was performed on a Bio-Rad iCycler iQ real-time detection apparatus (Bio-Rad Laboratories, Hercules, CA) using a FastStart SYBR Green Master (Roche Diagnostics GmbH, Manheim, Germany). The parameters of PCR amplification were: 10 sec at 94°C, 20 sec at 60°C and 30 seconds at 72°C, for 45 cycles. The identity of each product was established by DNA sequence analysis [29] and the specificity of PCR reactions was confirmed by melting curve analysis of the products and by size verification of the amplicon in a conventional agarose gel.

Real-time PCR data were expressed as the fold change of the target gene expression relative to the geometric mean (g.m.) mRNA expression of the housekeeping genes in each sample, as described by Vandesompele et al. [41]. The fold change in gene expression was calculated by the formula: , where C_T _is the threshold cycle, calculated by the iCycler software, ΔC_T _= (C_T_target gene – C_T_g.m.reference genes) and ΔΔC_T _= (ΔC_T _test sample - ΔC_T _control sample). A pool of uterine cDNAs from untreated, ovariectomized mice, was used as a control sample throughout the study and the ratio of the target gene mRNA/geometric mean of the four reference genes mRNA in this control sample was designated as 1. Each assay was performed in triplicate and negative controls were run for every assay.

### Data analysis

All values are expressed as the mean ± S.E.M; *n *represents the number of mice used. Statistical procedures included one-way ANOVA followed by Dunnett's test for multiple comparisons and Student's unpaired *t *test to compare the means of two groups using GRAPHPAD PRISM 4. Statistical significance was accepted when *P *< 0.05.

## Results

**Figure 1:**
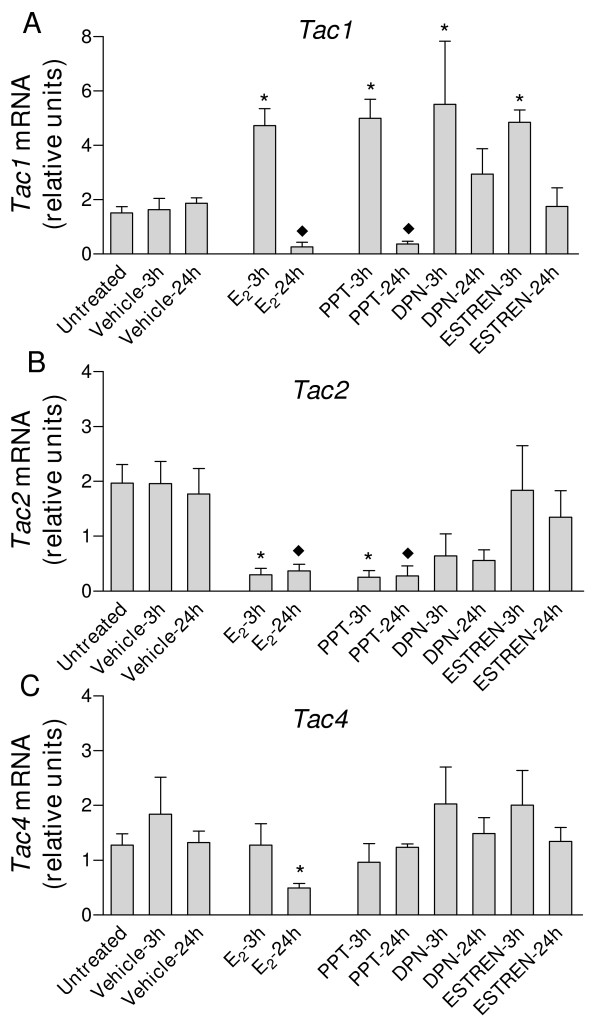
**Real-time quantitative PCR analysis of A) *Tac1*; B) *Tac2 *and C) *Tac4 *expression in uterine cDNA from mice treated with E_2_, propylpyrazole triol (PPT), diarylpropionitrile (DPN)or estren**. Uteri were collected from ovariectomized mice untreated (control) or treated for 3 or 24 h with E_2 _(1 μg/mouse) PPT (75 μg/mouse) DPN (100 μg/mouse) estren (300 μg/mouse) or the corresponding vehicle. Values are expressed as the fold change of each target-gene expression, relative to the geometric mean mRNA expression of 4 housekeeping genes. Each bar represents the mean of uterine cDNA samples from 5–10 different mice, with S.E.M. shown by vertical lines. *P < 0.05, significant difference versus mRNA levels in uteri from ovariectomized mice treated for 3 h with vehicle; ^♦^*P *< 0.05, significant difference versus mRNA levels in uteri from ovariectomized mice treated for 24 h with vehicle; one-way ANOVA.

**Figure 2:**
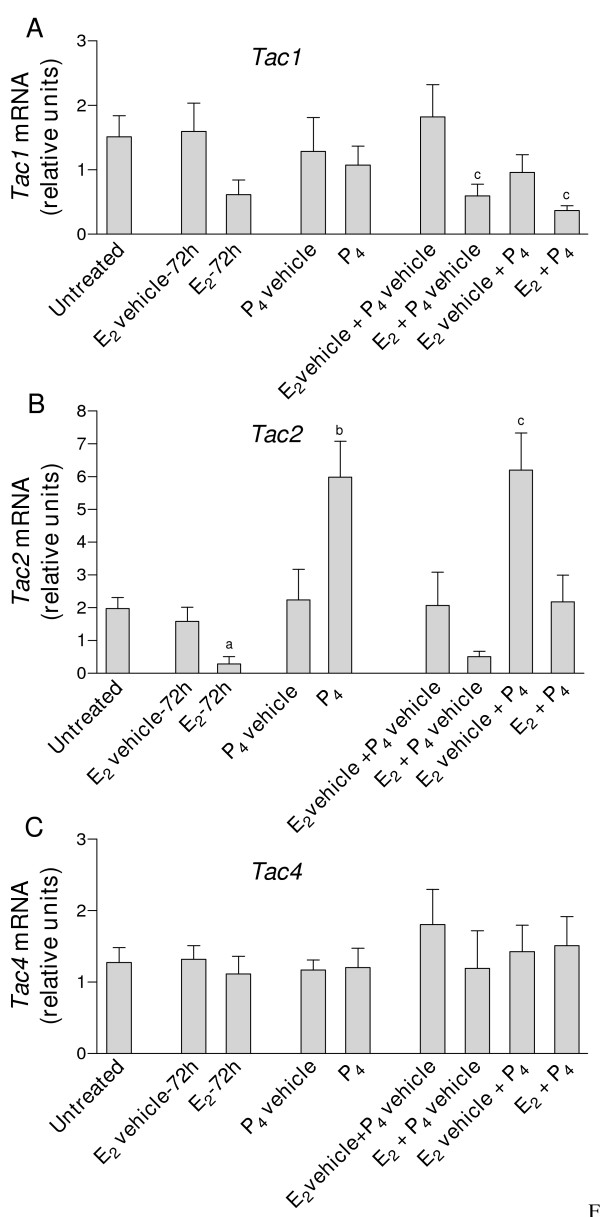
**Real-time quantitative PCR analysis of A) *Tac1*; B) *Tac2 *and C) *Tac4 *expression in uterine cDNA from mice treated with E_2 _and P_4_, alone or in combination**. Uteri were collected from ovariectomized mice untreated (control) or treated with E_2 _(1 μg/mouse, 72 h) or its vehicle, P_4 _(2 mg/mouse per day for 2 days) or its vehicle, and E_2 _+ P_4 _or the corresponding vehicles. Values are expressed as the fold change of each target-gene expression, relative to the geometric mean mRNA expression of 4 housekeeping genes. Each bar represents the mean of uterine cDNA samples from 5–10 different mice, with S.E.M. shown by vertical lines. ^a^*P *< 0.05, E_2 _vs. E_2 _vehicle, unpaired *t *test; ^b^*P *< 0.05, P_4 _vs. P_4 _vehicle, unpaired *t *test; ^c^*P *< 0.05, E_2 _+ P_4_, E_2 _+ P_4 _vehicle and E_2 _vehicle + P_4 _vs. E_2 _vehicle + P_4 _vehicle, one-way ANOVA.

**Figure 3:**
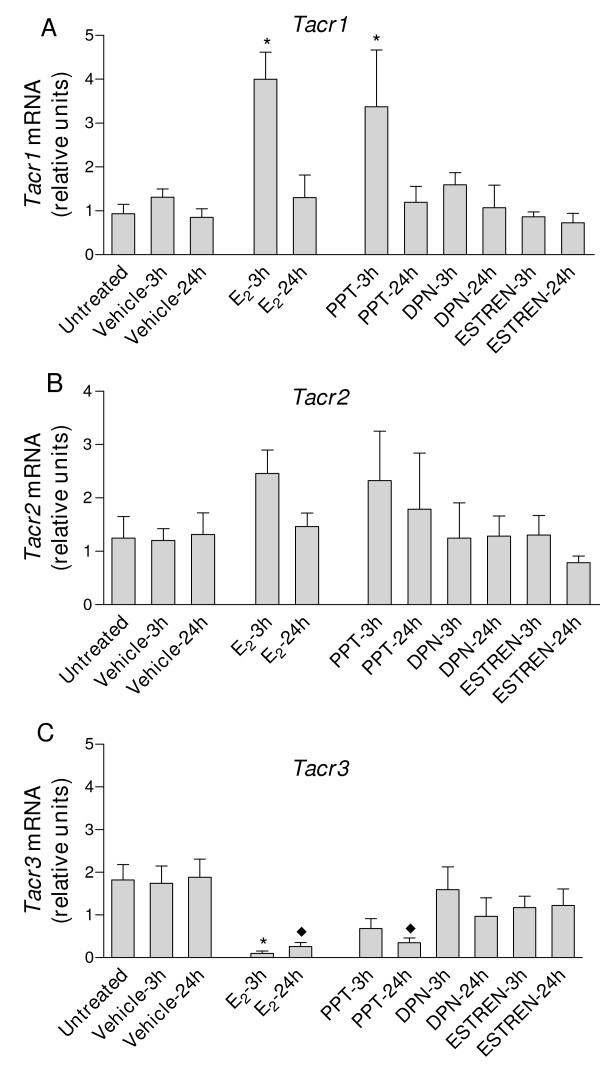
**Real-time quantitative PCR analysis of A) *Tacr1*; B) *Tacr2 *and C) *Tacr3 *in uterine cDNA from mice treated with E_2_, propylpyrazole triol (PPT), diarylpropionitrile (DPN)or estren**. Uteri were collected from ovariectomized mice untreated (control) or treated for 3 or 24 h with E_2 _(1 μg/mouse) PPT (75 μg/mouse) DPN (100 μg/mouse) estren (300 μg/mouse) or the corresponding vehicle. Values are expressed as the fold change of each target-gene expression relative to the geometric mean mRNA expression of 4 housekeeping genes. Each bar represents the mean of uterine cDNA samples from at least five different mice, with SEM shown by vertical lines. *P < 0.05, significant difference versus mRNA levels in uteri from ovariectomized mice treated for 3 h with vehicle; ^♦^*P *< 0.05, significant difference versus mRNA levels in uteri from ovariectomized mice treated for 24 h with vehicle; one-way ANOVA.

**Figure 4:**
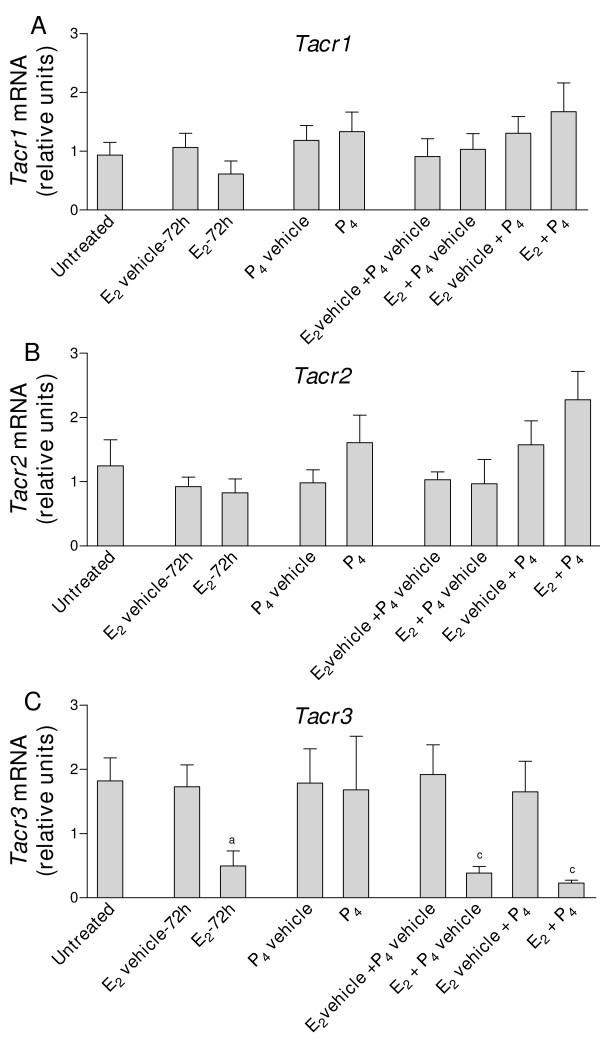
**Real-time quantitative PCR analysis of A) *Tacr1*; B) *Tacr2 *and C) *Tacr3 *expression in uterine cDNA from mice treated with E_2 _and P_4_, alone or in combination**. Uteri were collected from ovariectomized mice untreated (control) or treated with E_2 _(1 μg/mouse, 72 h) or its vehicle, P_4 _(2 mg/mouse per day for 2 days) or its vehicle, and E_2 _+ P_4 _or the corresponding vehicles. Values are expressed as the fold change of each target-gene expression, relative to the geometric mean mRNA expression of 4 housekeeping genes. Each bar represents the mean of uterine cDNA samples from 5–10 different mice, with S.E.M. shown by vertical lines. ^a^*P *< 0.05, E_2 _vs. E_2 _vehicle, unpaired *t *test; ^c^*P *< 0.05, E_2 _+ P_4_, E_2 _+ P_4 _vehicle and E_2 _vehicle + P_4 _vs. E_2 _vehicle + P_4 _vehicle, one-way ANOVA.

The transcripts for *Tac1*, *Tac2*, *Tac4*, *Tacr1*, *Tacr2 *and *Tacr3 *were detected in uteri from ovariectomized mice. In all experiments and with all target genes, mRNA expression values were similar in untreated and vehicle-treated mice (Fig. 1, Fig. 2, Fig. 3 and Fig. 4).

### Effects on tachykinin gene expression

*Tac1 *expression was increased 3-fold in uterine cDNA from mice treated with E_2 _for 3 h, compared with vehicle-treated (3 h) mice (Fig. 1A). PPT, DPN and estren also increased the *Tac1 *transcript 3 h after their administration (Fig. 1A). At 24 h, E_2 _decreased 7-fold the expression of *Tac1 *compared to the respective vehicle controls (Fig. 1A). Treatment with PPT for 24 h caused a 5-fold decrease in *Tac1 *mRNA while DNP and estren had no significant effects (Fig 1A). *Tac1 *mRNA levels remained low in mice after 72 h treatment with E_2 _(Fig. 2A). P_4 _was without effect and did not influence the E_2_-induced decrease in *Tac1 *expression (Fig. 2A).

Treatment with E_2 _for 3 or 24 h caused large decreases in *Tac2 *expression, compared with vehicle-treated mice (Fig. 1B). The decrease was still significant at 72 h (P < 0.05, see Fig. 2B). PPT decreased *Tac2 *mRNA after 3 and 24 h treatment, while neither DPN nor estren had significant effects (P > 0.05, Fig. 2B). *Tac2 *mRNA was increased 3-fold in mice treated with P_4 _(Fig. 2B); neither this increase nor the E_2_-induced decrease were seen in mice treated with both E_2 _and P_4 _(Fig. 2B).

*Tac4 *mRNA levels were decreased 3-fold in mice treated with E_2 _for 24 h, compared with uteri from corresponding vehicle-treated mice. As shown in Figs. 1C and 2C, none of the other treatments modified *Tac4 *expression.

### Effects on tachykinin receptor gene expression

*Tacr1 *expression was increased in mice treated for 3 h with E_2 _or PPT (Fig. 3A). After treatment with E_2 _or PPT for 24 h, the mRNA levels were similar to those observed in control, vehicle-treated mice. DPN and estren had no effects either at 3 or 24 h (Fig. 3A). P_4_, alone or in combination with E_2_, was without effect on *Tacr1 *levels (Fig. 4A).

Compared with uteri from control, vehicle-treated mice, E_2 _and P_4 _(alone or in combination), PPT, DPN and estren did not cause significant alterations in *Tacr2 *mRNA levels (P > 0.05, Figs. 3B and 4B).

*Tacr3 *expression was decreased in mice treated with E_2 _for 3 or 24 h and in mice treated with PPT for 24 h, compared with vehicle-treated mice (Fig. 3C). DPN and estren had no effect (Fig. 3C). *Tacr3 *mRNA levels remained low in uteri from mice treated with E_2 _for 72 h (Fig. 4C). P_4 _did not modify *Tacr3 *expression and did not influence the E_2_-induced decrease in the expression of *Tacr3 *(Fig. 4C).

## Discussion

The present study has shown that ovarian steroids tightly and differentially regulate the expression of tachykinin and tachykinin receptor genes in the mouse uteri. Since the uterus is often considered a model to investigate the effects of steroid hormones on gene transcription, this study represents the first analysis of the regulatory effects of E_2 _and P_4 _on the whole tachykinin family.

*Tac1 *mRNA and the tachykinin peptides encoded by this gene, predominantly SP and NKA, are widely expressed in neuronal and non-neuronal cells at both central and peripheral levels [3,10,20-22,26,29,32]. Several workers have investigated the effects of ovarian steroids on the expression of this gene and its protein products in the CNS, particularly in the hypothalamic-pituitary axis [18,20-22,32], often with conflicting results. Our findings show that *Tac1 *was regulated by E_2 _in a complex and time-dependent manner, with both stimulatory and inhibitory effects. The higher expression of *Tac1 *in ovariectomized animals, compared to non-ovariectomized mice [29], together with the observation that 24–72 h treatment with E_2 _causes a strong and long-lasting down-regulation of the *Tac1 *transcript clearly points to the importance of *Tac1 *regulation by estrogen. In contrast to E_2_, P_4 _had no effect on *Tac1 *expression nor did it influence the E_2_-induced decrease.

In the mouse uterus there is a predominance of ERα, while ERβ modulates some of its effects [4-6]. In our study, the E_2 _effect was mimicked by PPT, a highly selective agonist of ERα devoid of activity on ERβ [7,40]. In contrast DPN, a highly selective agonist of ERβ [7], or estren, a purported activator of non-genomic E_2 _signaling [8], were without effect. It thus seems likely that nuclear ERα mediates the inhibitory effect of E_2 _(after 24 h) on *Tac1 *expression.

Early physiological responses of the mouse uterus to acute E_2 _appear within the first 2–3 h while late responses are observed 16–24 h after E_2 _[4,5]. Consistent with these findings, 3 h treatment with E_2 _caused an increase in *Tac1 *mRNA levels. This early response was also observed in animals treated with PPT, DPN or estren. These findings suggest that early stimulation of *Tac1 *is mediated by both genomic and non-genomic E_2 _pathways.

The NK_1_R is the preferred receptor for SP, while NKA can also act as a potent agonist on NK_1_R [13-17]. Therefore, it was interesting to observe that treatment with E_2 _for 3 h caused a parallel increase in both *Tac1 *and *Tacr1 *mRNAs. *Tacr1 *gene expression was also increased in mice treated for 3 h with PPT revealing the participation of ERα. Since tachykinins are potent inflammatory mediators, the present findings may suggest that SP(NKA)-NK_1_R ligand-receptor pair could play a role in mediating rapid effects of E_2 _in the uterus, such as hyperemia, water imbibition and attraction of immune cells.

Treatment of ovariectomized mice with E_2_, PPT, DPN or P_4 _did not decrease NK_1_R gene expression. This finding was unexpected, as we previously found that the transcript for *Tacr1 *decreases by about 11-fold on day 17 of pregnancy, compared with its expression in uteri from non-pregnant mice [29]. The NK_1_R is regulated by many different mechanisms and it may be that near term, in addition to ovarian steroids, other hormonal, humoral, placental or neural influences may influence its expression.

The NK_2_R is the key tachykinin receptor mediating contractile responses to TKs in the near term uteri from all mammalian species studied [10,29,30]. Furthermore, the participation of the NK_2_R in mouse myometrial contractions is higher under conditions of estrogen dominance [29,39]. The expression of *Tacr2 *in uteri from ovariectomized mice was, however, unaffected by treatment with E_2 _or P_4_. This result is consistent with our previous observation that *Tacr2 *mRNA levels are similar in uteri from pregnant or non-pregnant mice and remain essentially constant during different hormonal stages [29]. Taken together, these findings suggest that, at least in the mouse uterus, the *Tacr2 *gene is not a direct target for ovarian steroids. The increased response in late pregnancy to agonists that act at the NK_2_R [29] may therefore reflect regulation at a posttranscriptional level.

NKB, together with SP, kisspeptin and dinorphin, is present in a group of hypothalamic neurons that also express estrogen and progesterone receptors [19,21,42,43]. NKB is also expressed at all main levels in the female genital tract [10,24-26,29-33] and its excessive secretion from the placenta may cause, in part, some of the symptoms of pre-eclampsia [24,25,31]. Recently, the genome sequence of a non-placental mammal, namely the platypus *Ornithorhynchus anatinus*, has become available [44] and by phylogenetic analysis, we found that the NKB encoding gene is present (Ensemble entry ENSOANG00000022075). This finding clearly shows that NKB plays additional roles in reproduction, apart from its participation in placental pathophysiology.

NKB is elevated in the human female hypothalamus after menopause [21]. Additionally, its expression in the rodent brain and in the rat uterus is increased by age and ovariectomy and decreased by E_2 _treatment [3,19,38,42]. These findings and our current observations showing that *Tac2 *mRNA is strongly repressed in uteri from young ovariectomized mice treated with E_2 _at all times of treatment indicate that at both central and peripheral levels, NKB over-expression is secondary to ovarian failure [19,38].

*Tac2 *inhibition was mediated by ERα because it was observed in mice treated for 3 or 24 h with PPT, but not with DPN or estren. In addition, our data show, to our knowledge for the first time, that P_4 _treatment up-regulated *Tac2 *gene expression in uteri from ovariectomized mice. The mechanism that mediates this P_4 _effect remains unclear, as in ovariectomized mice, the expression of PRs fall as a consequence of the lack of E_2 _[7]. Although the presence of a basal population of nuclear PRs cannot be excluded, the effects on *Tac2 *expression may more probably be mediated by activation of membrane-bound P_4 _receptors distinct from PRs. Several P_4 _membrane receptor candidates are present in uteri from ovariectomized mice and the expression of some of them is increased by P_4 _[9]. Up-regulation by P_4 _would permit a local increase in *Tac2 *expression following ovulation or in the case of copulation and successful fertilization. These data are in agreement with our previous observations showing that the highest *Tac2 *levels were found in the mouse uterus around the time of implantation and that these were strongly decreased at late pregnancy [29].

At the peripheral level the role of NK_3_R, like that of its preferred ligand NKB, remains poorly understood [10,31,32,36,45]. The present data show that E_2 _strongly reduces *Tacr3 *expression in uteri from ovariectomized mice. The E_2_-induced responses were mimicked by PPT but not by DPN or estren, providing the first evidence, to our knowledge, that the effects of E_2 _on this receptor are mediated by ERα. The observation that E_2 _caused a rapid and concomitant down-regulation of *Tac2 *and *Tacr3 *expression supports the existence of an important link between E_2 _and the NK_3_R/NKB activation pathway [38]. Moreover, the tight regulation of both genes strongly argues for a role of NKB, acting through the NK_3_R, in mediating some of the effects of E_2 _and P_4 _on uterine function [34,38]. In this context, a recent report has established a correlation between familial hypogonadotropic hypogonadism and mutations in the human genes *TAC3 *and *TACR3*·[23] providing clear evidence for the importance of NKB and the NK_3_R in reproduction.

The *Tac4 *gene is expressed in the placenta, the ovary, the uterus, and also in oocytes and blastocyst-stage embryos [12,29,32,37]. In the mouse uteri, HK caused myometrial contractions that are mediated by the NK_1_R and are decreased at late pregnancy [39]. Apart from its effects on myometrial contractility, the physiological role of HK at the reproductive level remains poorly understood and its direct regulation by ovarian steroids has not been studied. The present data show that the *Tac4 *gene is also a target of E_2 _and suggest that *Tac4 *expression is down-regulated by E_2 _surges, as those observed in mammals before ovulation or near labor.

## Conclusion

This study suggests that uterine tachykinins and tachykinin receptors are important targets of ovarian steroids and particularly of E_2_, acting at ERα. The whole tachykinergic system appears involved in the regulation of reproductive functions.

## Abbreviations

SP: substance P; NKA: neurokinin A; NKB: neurokinin B; HK1: hemokinin 1; TK: tachykinin; NK_1_R: NK_1 _receptor; NK_2_R: NK_2 _receptor; NK_3_R: NK_3 _receptor; E_2_: estrogen; ERα: estrogen receptor α; ERβ: estrogen receptor β; PPT: propylpyrazole triol; DPN: diarylpropionitrile; P_4_: progesterone; PR: progesterone receptor; CNS: central nervous system.

## Competing interests

The authors declare that they have no competing interests.

## Authors' contributions

FMP carried out PCR experiments and helped to write the manuscript. COP performed ovariectomy, ovarian steroid treatments and collected uterine samples. EP performed the statistical analysis. JNP and LC wrote the manuscript. FMP and LC designed the study. All authors read and approved the final manuscript.
